# Targeting the Interplay of Autophagy and ROS for Cancer Therapy: An Updated Overview on Phytochemicals

**DOI:** 10.3390/ph16010092

**Published:** 2023-01-08

**Authors:** Lixia Dong, Jingqiu He, Li Luo, Kui Wang

**Affiliations:** 1 West China School of Basic Medical Sciences and Forensic Medicine, Sichuan University, Chengdu 610041, China; 2 Center for Reproductive Medicine, Department of Gynecology and Obstetrics, West China Second University Hospital, Sichuan University, Chengdu 610041, China; 3 Key Laboratory of Birth Defects and Related Diseases of Women and Children, Sichuan University, Ministry of Education, Chengdu 610041, China

**Keywords:** ROS, autophagy, cancer, phytochemicals

## Abstract

Autophagy is an evolutionarily conserved self-degradation system that recycles cellular components and damaged organelles, which is critical for the maintenance of cellular homeostasis. Intracellular reactive oxygen species (ROS) are short-lived molecules containing unpaired electrons that are formed by the partial reduction of molecular oxygen. It is widely known that autophagy and ROS can regulate each other to influence the progression of cancer. Recently, due to the wide potent anti-cancer effects with minimal side effects, phytochemicals, especially those that can modulate ROS and autophagy, have attracted great interest of researchers. In this review, we afford an overview of the complex regulatory relationship between autophagy and ROS in cancer, with an emphasis on phytochemicals that regulate ROS and autophagy for cancer therapy. We also discuss the effects of ROS/autophagy inhibitors on the anti-cancer effects of phytochemicals, and the challenges associated with harnessing the regulation potential on ROS and autophagy of phytochemicals for cancer therapy.

## 1. Introduction

As early as 1963, Christian de Duve found that heterogenic intracellular cargoes could be transported to lysosomes for degradation and named this phenomenon “autophagy” [1]. The detailed regulatory mechanism of autophagy has been discovered and displayed since the 1990s. Today, it is well known that autophagy is a cellular self-degradative progress that delivers cytosolic components and damaged organelles to the lysosomes for hydrolytic enzyme-mediated digestion and subsequently recycles the generated catabolites to support anabolic metabolism. The autophagy progress is evolutionarily conserved in eukaryotes, which involves many complex molecular pathways and various ATG proteins encoded by more than 40 genes [2]. Under starvation, hypoxia, and other stress conditions, ATG proteins associated with autophagy initiation will assemble on the endoplasmic reticulum (ER) to form a double membrane structure, named a phagophore. Next, the phagophore surrounds cellular macromolecules in a selective or nonselective way and expands constantly to take the shape of a closed autophagosome. The autophagosome vesicle can then be transported to and fused with the lysosome to form the autolysosome, in which the lysosomal hydrolase will then degrade internal components for catabolite reutilization [3]. Generally, cells often perform basal level autophagy to maintain intracellular homeostasis; however, overactivation of excessive autophagy may disturb this homeostasis, cause cell damage, and even result in cell death. Disorder of autophagy has been reported to be involved in different pathologies, such as neurodegenerative disorders, metabolic syndromes, and cancers [4]. When it comes to cancer, it is currently believed that autophagy operates as a tumor suppressor in healthy cells but promotes cancer development in transformed cells [5,6,7].

Aerobic respiration is a highly efficient form of energy production that can support the development of eukaryotes. However, ROS, the byproduct of aerobic respiration, can cause damage to biomacromolecules such as lipids, nucleic acids, and proteins, thereby changing their functions and causing oxidative stress. However, recent studies demonstrated that ROS can also act as a signaling molecule to regulate various signaling pathways to maintain normal development of cells [8]. It has been widely accepted that ROS can be both beneficial and lethal for cancer cells. On the one hand, ROS can reversibly oxidize cysteine residues of many proteins to regulate their function and promote cancer cell proliferation, metabolism, and metastasis [9]. On the other hand, high levels of ROS can suppress cancer development by provoking oxidative stress, inducing cell death and senescence [10]. Notably, ROS can induce autophagy by modulating different signaling pathways, either inhibiting or enhancing the development of cancers. For instance, autophagy can specifically eliminate damaged mitochondria to reduce ROS accumulation, thus inhibiting the deleterious effect of ROS on DNA and suppress cancer initiation. In the development of cancer, one of the effects of autophagy is to remove excessive ROS caused by hypoxia or damaged organelles, thus maintaining the survival of cancer cells [11]. What we can conclude is that the mutual regulation between autophagy and ROS complicates their roles in cancer.

Cancer is a public health problem worldwide because of its high morbidity and mortality [12]. Traditional therapies for cancer, such as chemotherapy and radiotherapy, have obvious adverse treatment-related effects. Chemotherapy is the main treatment approach for numerous cancers because it can influence all the stages of cell development by regulating various signaling pathways. However, the therapeutic effect of traditional chemical drugs varies depending on the types and stages of tumors. In addition, tumors can become resistant to chemical drugs in the later stages of treatment. Meanwhile, chemotherapy can seriously reduce a patient’s quality of life because of its highly toxic effects [13,14,15]. Therefore, it is urgent to find new anti-cancer drugs with lower toxicity and higher therapeutic effect. Phytochemicals, extracted from various medicinal plants, or their synthetic analogues, can prevent tumor proliferation, invasion, and migration by modulating various signaling pathways [16,17,18]. Furthermore, some natural bioactive products coming from food exhibit relatively low toxicity and potent chemo-preventive properties [19]. Thus, phytochemicals have attracted much attention for cancer treatment in recent years [20].

Various phytochemicals have been found to possess potent anti-cancer effects, but their anti-cancer mechanism is unclear, which limits their clinical application. Nowadays, studies have increasingly paid attention to exploring the anti-cancer mechanism of phytochemicals, among them, the regulation of autophagy and ROS have been widely investigated. In this review, we expounded the interplay between autophagy and ROS, and introduced some phytochemicals that can treat cancer by modulating autophagy and ROS.

## 2. The Process of Autophagy and Its Role in Cancer

**Figure 1:**
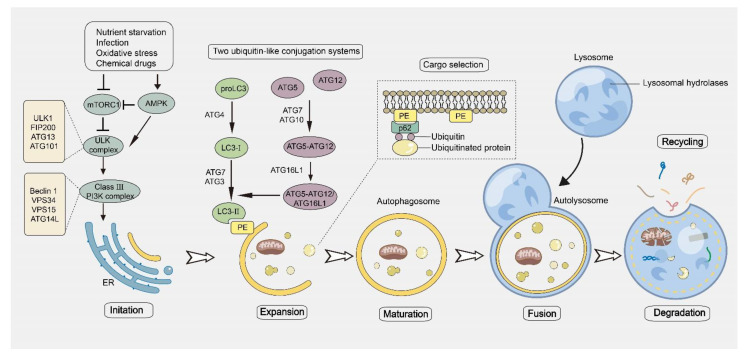
The progress of autophagy. Various stress conditions such as nutrient starvation, infection, oxidative stress, and chemical drugs lead to an increase in mTORC1 suppression and AMPK activation. This can activate the ULK1 complex through a number of phosphorylation cascades. Subsequently, phagophores are formed at the autophagy initiation site of the endoplasmic reticulum, and the phagophores envelop substrates and extend continuingly until forming a double-layered vesicle named autophagosomes with the participation of various autophagy proteins. Finally, intact autophagosomes fuse with the lysosomes to form autolysosomes where the substrates will be degraded by hydrolytic enzymes.

Classic autophagy is a multi-step process that involves the consecutive and selective aggregation and interaction of various ATG proteins. When the cells are subjected to stimulation, the first protein to respond is the ULK1 (Unc-51-like kinase 1). Signal molecules can activate ULK1, which can phosphorylate ATG13 and FIP200 to form the ULK1 complex (consisting of ULK1, ATG13, and ATG101). The ULK1 complex is anchored to the pre-autophagosomal structure (PAS, a special ER domain, also named the omegasome) to form the PAS scaffold complex (Figure 1). The class III phosphatidylinositol 3-kinase (PI3K) complex, ATG9A system, ATG12-conjugation system, and LC3-conjugation system are then engaged hierarchically to the PAS by the scaffold FIP200 [21,22]. Following the gathering of the ULK1 complex, it facilitates the activation of the autophagy-specific class III PI3K complex (including PI3K, VPS15, Beclin 1, and ATG14L) by phosphorylating Beclin 1. Subsequently, PI3K will phosphorylate phosphatidylinositol (PI) to form phosphatidylinositol 3-phosphate (PtdIns(3)P or PI3P) at the omegasome [23,24,25]. Next, WD-repeat domain phosphoinositide-interacting proteins (WIPIs) and double FYVE domain-containing protein 1 (DFCP1) successively assemble at the omegasome via their PI3P-binding domain to form phagophores. WIPI2 can interact with ATG16L to recruit the ATG12-ATG5-ATG16L complex to the PSA [26,27,28,29]. The ATG12-ATG5-ATG16L complex is one of the ubiquitin-like conjugation systems for autophagosome formation, and another is the LC3 conjugation system. Neonatal LC3 is a soluble protein that can be catalyzed by ATG4 to expose a glycine residue [30,31]. The decorated LC3, also named LC3-I, is bound to E1-like enzyme ATG7, and is subsequently transferred to the E2-like enzyme ATG3. LC3-I is then conjugated to phosphatidylethanolamine (PE) by ATG3 to form membrane-bound LC3-II [32,33], which serves as a well-known autophagosome marker. The conjugation of LC3-I with PE can also be enhanced by the ATG12-ATG5-ATG16L complex, which acts as the E3-like enzyme. LC3-II is embedded in the membrane of phagophores to promote membrane extension (Figure 1). In addition, autophagy receptors can interact with LC3-II via the LC3-binding motif to transfer special cargos to the LC3-II-containing phagophores [34,35,36]. Up to now, several autophagy receptors, including p62, chaperonin containing TCP1 subunit 2 (CCT2), nuclear dot protein 52 kDa (NDP52), optineurin (OPTN), BNIP3-like (BNIP3L)/ NIX, and neighbor of BRCA1 gene 1 (NBR1), have been identified, which mediate the selective autophagy of cargos. The phagophores then extend continuingly until forming a sealed double-layered vesicle, called the autophagosome. During this process, Atg2 and Atg9 can transport lipids from the cytoplasmic leaflet of the ER or the cytoplasmic to omegasome, thereby providing materials and enabling the expansion of the autolysosome [37,38,39,40,41]. The intact autophagosome can fuse with the lysosome to form the autolysosome, where the substrates will be degraded by hydrolytic enzymes (Figure 1).

Because autophagy can maintain homeostasis against internal and external stresses, it is important for cells to maintain a basal level of autophagy. Disorders of autophagy are associated with many diseases, such as cancer. Autophagy plays an indispensable and double-edged sword role in cancer. In the early stages of tumor development, autophagy is commonly considered to be a tumor-suppressive, owing to its ability to clear damaged intracellular component and maintain genetic stability to prevent tumorigenesis [42,43,44]. For instance, the Beclin 1 haploid-insufficient mouse was more likely to develop a malignant tumor [45,46]. Mice with ATG5 homozygote deletion have a higher probability of live tumors [47]. These evidences prove that autophagy could suppress tumorigenesis. However, the aggregation of p62 has been detected in gastrointestinal cancer, hepatocellular carcinoma, breast cancer, and lung adenocarcinoma [48,49,50,51,52]. In Kras-driven lung cancer models, a deficiency in Atg7 decreases the amino acid substrate supply to mitochondria, resulting in extreme fatty acid oxidation, which consumes lipid stores and promotes energy crisis [53]. Therefore, autophagy can also promote tumor cell survival and growth by providing metabolic substrates.

## 3. The Interplay between Autophagy and ROS in Cancer

### 3.1. The Regulation of Autophagy Machinery

Autophagy in cancer can be precisely regulated by a serious of signaling molecules and regulatory pathways in response to intracellular and extracellular stimuli, such as starvation, oxidative stress, ER stress, and alteration of the AMP/ATP ratio. Mechanistic target of rapamycin (mTOR) and AMP-activated protein kinase (AMPK) are two primary regulators for initiating autophagy.

mTOR, an evolutionarily conserved serine/threonine kinase, can be regulated by nutritional status, growth factor, and stress signals. mTOR contains two different complexes, named mTORC1 and mTORC2, out of which mTORC1 is sensitive to rapamycin and has the ability to impact cell growth and proliferation by regulating anabolic and catabolic metabolism, including autophagy [54]. mTORC1 can negatively regulate autophagy. When mTORC1 is activated, it connects to the ULK1 complex by Raptor and inhibits the kinase activity of the ULK1 complex by phosphorylating ULK1 at Ser758 and Ser638, as well as ATG13 at Ser258 [55,56,57]. On the contrary, inactivated mTORC1 will dissociate from the ULK1 complex, and then phosphatases, such as protein phosphatase 2A (PP2A) and protein phosphatase 1D magnesium-dependent delta isoform (PPM1D), relieve the phosphorylation status of ULK1 and ATG13 [58,59,60]. ULK1 then undergoes autophosphorylation at Thr180 and subsequently phosphorylates ATG13, FIP200, and ATG101, and eventually, the ULK1 complex can be activated and then transferred to phagophores to support the initiation of autophagy [61]. In addition to direct regulation, mTORC1 can also indirectly intercept autophagy initiation by inhibiting ULK1 stability and activity. In detail, mTORC1 can suppress the activation of TNF receptor-associated factor 6 (TRAF6) an E3 ubiquitin ligase, through phosphorylating AMBRA1, and thus prevent ubiquitination of ULK1 [62].

In addition, mTORC1 can be regulated by various upstream signaling molecules. When G protein-coupled receptors (GPCRs) or receptor tyrosine kinases (RTKs) are phosphorylated and activated, the regulatory subunit p85 is recruited to the vicinity of the plasma membrane to activate PI3K. The activated PI3K can catalyze the phosphorylation of phosphatidylinositol-4,5-bisphosphate (PIP2) to form phosphatidylinositol-3,4,5-triphosphate (PIP3). PIP3 will promote the recruitment of protein kinase 3-phosphoinositidedependent protein kinase-1 (PDK1) and AKT. Subsequently, PDK1 can activate AKT by phosphorylation at Ser308, pleading to the heterodimerization and inhibition of tuberous sclerosis 1 (TSC1) and TSC2. Inactivation of the TSC1-TSC2 complex holds RHEB in its GTP-bound state, thereby activating its GTPase activity to enable mTORC1 activation. AKT can also phosphorylate and dissociate the mTORC1 inhibitor PRAS40 from Raptor to activate mTORC1 [63,64,65]. The regulation autophagy by AKT has also been reported to be mTOR-independent. In detail, AKT can phosphorylate and inactivate FOXOs, which are the transcription factors in charge of the transcription of some autophagy-related genes, including Beclin 1, ATG12, and GABARAPL1 [66]. Notably, the tumor suppressor phosphatase and tensin homolog (PTEN) can promote the conversion of PIP3 to PIP2 to inhibit AKT, thereby favoring the initiation of autophagy [67,68].

Autophagy can also be regulated by AMPK, which senses the cellular energy status through the alteration of the AMP/ATP ratio. AMPK can be phosphorylated at Thr172 and activated by AMP or the upstream kinases such as the liver kinase B1 (LKB1), calcium/calmodulin-dependent protein kinase kinase 2 (CAMKK2), and the MAPKKK family [69,70,71,72]. Upon energy starvation, activated AMPK can induce autophagy by increasing ULK1 activity through direct phosphorylation at Ser317, Ser777, and Ser555 [73]. Furthermore, AMPK can negatively regulate mTORC1 activity through phosphorylating and inactivating Raptor at Ser722 and Ser792 or activating TSC2 by phosphorylating Thr1227 and Ser1345 [74,75], thereby initiating autophagy.

In addition, several transcriptional factors have been reported to regulate autophagy in cancer. Transcription factor EB (TFEB), the regulator of lysosomal biogenesis, can enter the nucleus to promote transcription of autophagy-related genes, such as ATG16, ATG4, p62, WIPI proteins, LC3, ULK1, and several cathepsins [76,77]. Hypoxia-inducible factor 1 (HIF-1), which is affected by the extent of hypoxia, promotes the transcription of Bcl-2 interacting protein 3 (BNIP3) and BNIP3L, which competitively interrupts the Bcl-2-Beclin-1 complex, resulting in the association of Beclin-1 with PI3K for autophagy activation [78,79]. Severe hypoxia also activates transcription factor 4 (ATF4), which can translocate into the nucleus to promote the transcription of ATG5, ULK1, and LC3, thereby promoting the autophagy process [80,81,82]. In addition, p53 has the function of regulating autophagy. Nuclear p53 can induce autophagy by transcriptionally upregulating the expression of AMPK, damage-regulated autophagy modulators (DRAMs), sestrin 1 and 2, and DAPK1 (death-associated protein kinase 1). Meanwhile, the transcription of ATG4, ATG7, ATG10, and ULK1 can also be promoted by nuclear p53. However, other studies showed that the cytoplasmic p53 can inhibit autophagy through directly interacting with FIP200 [83,84,85]. Similarly, NF-κB also play a dual role in the regulation of autophagy. It has been demonstrated NF-κΒ can induce autophagy by directly promoting the transcription of ATG genes, such as Beclin 1, ATG5, and LC3, or can inhibit autophagy by increasing the expression of autophagy repressors such as Bcl-2 family members and PTEN/mTOR or suppressing the expression of autophagy inducers like BNIP3 and JNK1 [86,87,88].

### 3.2. The Relationship between Autophagy and ROS in Cancer

**Figure 2:**
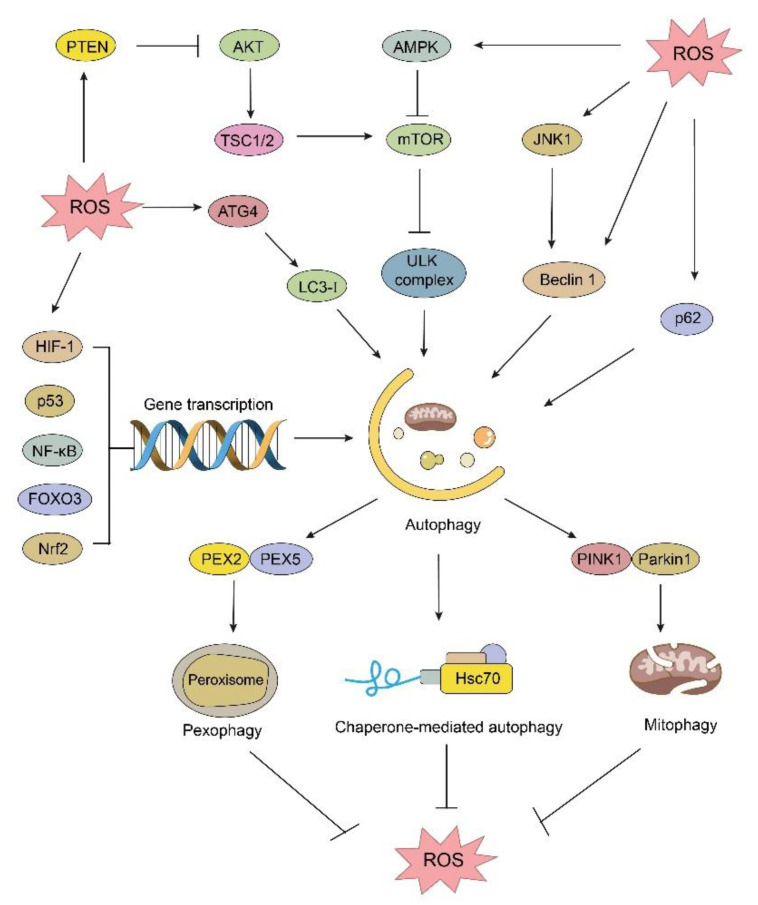
Relationship between ROS and autophagy. ROS can regulate autophagy in different ways. ATG4 and p62 can be oxidized directly by ROS to induce autophagy; ROS can activate the AMPK signaling cascade or inhibit the mTOR pathway, resulting in autophagy initiation; transcription factors such as Nrf2 can also be activated by ROS, which will promote the transcription of various ATG proteins. Autophagy can also decrease the generation of ROS by pexophagy, mitophagy, and CAM.

Basal levels of ROS play an important role in regulating cell proliferation, immune response, and differentiation, but superfluous ROS cause damage to biomolecules, such as proteins, lipids, and nucleic acids, resulting in various diseases including cancer. Autophagy is one of the most crucial processes that can be regulated by ROS, serving as a signal molecule [89,90]. Firstly, numerous studies suggest autophagy can be regulated by ROS through mTOR-dependent pathways. On the one hand, high levels of ROS can promote the inactivation of PTEN by direct oxidation [91], which can increase PIP3 to activate AKT and inhibit mTORC1 signaling [92,93]. On the other hand, the activation of mTOR can be impacted by MAPK subfamilies, such as c-Jun N-terminal kinase (JNK), p38, and extracellular signal-regulated kinase (ERK), which could be regulated by ROS. It has been reported that accumulation of ROS can regulate autophagy by the MAPK/JNK/mTOR pathway [94,95,96]. Otherwise, MAPK/JNK can modulate autophagy by directly phosphorylating ULK1 or promoting the transcription of autophagy gene like Beclin 1 [97]. Secondly, ROS can also activate AMPK, which releases the inhibition of autophagy by directly phosphorylating and activating ULK1 or inhibiting mTORC1 [90,98]. It has been proven that ROS can inhibit mTOR or enhance AMPK signal pathways to induce autophagy in various cancer cells, impacting cancer progression [99,100]. In addition, transcription factors that modulate the expression of ATG genes, such as TFEB, HIF-1α, p53, NF-κB, FOXO3, ATF4, and Nrf2, can be activated by increased levels of ROS. For example, ROS was reported to directly oxidize the Cys211 of TFEB. Cys211 oxidation inhibited the association of TFEB with Rag GTPases and promoted the nuclear translocation of TFEB, which in turn led to an increased gene expression level in the autophagy-lysosome system [101]. Furthermore, under some circumstances, high levels of ROS can cause mitochondrial dysfunction, leading to the selective removal of damaged mitochondria (called mitophagy) [102,103]. Furthermore, it has been proven that ER stress can be activated to trigger the unfolded protein response (UPR) in response to ROS accumulation, and autophagy can be induced by the ROS-mediated ER stress [104,105]. Elevated levels of ROS are thought to induce autophagy by activating these transcription factors, resulting in the degradation of damaged mitochondria, thereby maintaining homeostasis and promoting cancer cell growth (Figure 2).

Apart from the mechanisms mentioned above, ROS can regulate autophagy by directly oxidizing autophagy-related proteins. For example, ATG4 is inactivated by ROS-mediated oxidization of a critical catalytic site, Cys78. Oxidized ATG4 loses its delipidation ability for LC3, thereby increasing LC3 lipidation for autophagosome formation and autophagy induction [106]. Recently, it was proposed that autophagy receptor p62 was sensitive to oxidative stress, and its Cys105 and Cys113 can be oxidized by ROS to form p62 oligomers. Oxidized P62 oligomers were capable of engaging substrates to nascent autophagosomes and assisting biogenesis of autophagosomes [107,108]. Recently, it has been proven that increased expression of ATG4 and p62 can promote the growth of cancer cells [109,110,111] (Figure 2).

In addition to ROS-mediated regulation of autophagy, autophagy can in turn scavenge ROS and oxidized proteins through lysosome-dependent pathway during oxidative stress, leading to the maintenance of redox homeostasis of cancer cells. Mitochondria is the primary place of oxidative phosphorylation in eukaryotic cells, and the vast majority of intracellular ROS are derived from the electron transport chain (ETC) of the inner mitochondrial membrane (mtROS) under normal conditions [112,113]. However, defective mitochondria can bring devastation to cancer cell by producing a large amount of ROS, and the dysregulated ROS-generating mitochondria can be obliterated by mitophagy to resist mtROS-mediated oxidative stress [114,115]. Peroxisomes are involved in fatty acid beta-oxidation (FAO) and catabolic reactions of some lipids such as oleic acid, which can contribute to the production of ROS. Meanwhile, peroxisomes can eliminate ROS by generating various antioxidant enzymes. Thus, the selective degradation of ROS-generating or damaged peroxisomes can reduce the generation of ROS and contributes to the intracellular oxidative homeostasis [116,117]. Moreover, chaperone-mediated autophagy (CMA) can be activated by oxidative stress to maintain redox homeostasis [118]. Oxidized protein aggregates that cannot be degraded by proteasomal pathway can be recognized by the chaperone protein Heat-shock cognate protein 70 (Hsc70), to form a chaperone-substrate complex together with co-chaperones (such as 90 KD heat shock protein, Hsp90). This complex can then connect with lysosome-associated membrane protein type 2A (LAMP2A) through which the cargo is transported to lysosome for digestion [11,119]. It has been firmly established that the Nrf2-Kelch-like ECH-associated protein 1 (Keap1)-antioxidant response element (ARE) pathway is an adaptive cellular response conferring protection against oxidative and xenobiotic stress [120]. Phosphorylated p62 could directly bind with Keap1, then interrupt the Nrf2-Keap1 interaction and segregate Keap1 into autophagosomes, resulting in Nrf2 stabilization. Nrf2 is then translocated into the nucleus to promote the transcription of antioxidant genes [121,122] (Figure 2).

## 4. Phytochemicals Targeting ROS and Autophagy for Cancer Therapy

Phytochemicals are valuable sources of drug discovery for multitudinous diseases including cancer due to their various sizes, complex structures, and low toxicity. Notably, great advances have been made in uncovering phytochemicals that can target the interplay of ROS and autophagy. Considering this, we introduce the phytochemicals that are identified to target the interplay of ROS and autophagy for cancer therapy over the recent years (Table 1).

### 4.1. Celastrol

Celastrol (also known as tripterine), a widely studied quinine methide triterpenoid, is deemed to be the most active and prospective component of *Tripterygium wilfordii Hook F* (TWHF), which has a long history of treating rheumatoid arthritis (RA) [166,167]. Numerous studies have demonstrated that celastrol has potent anti-inflammation and anti-diabetes activities. For example, celastrol mitigated the inflammation of colitis by decreasing the colon myeloperoxidase concentration and colonic pro-inflammatory cytokines or inactivating the NLRP3 inflammasomes to reduce IL-1β secretion [168,169]. Meanwhile, its anti-cancer potential has aroused great interest recently. Treatment with celastrol inhibits the growth and proliferation of various cancer cells, such as colorectal, gastric, breast, lung, pancreatic, skin, prostate, and blood cancer cells. Mechanistically, celastrol can induce apoptosis, promote cell cycle arrest, and suppress metastasis and angiogenesis, etc. [170]. According to a recent paradigm, celastrol has also been shown to exhibit anti-cancer effects by inducing autophagy through ROS accumulation. For instance, Li et al. demonstrated that celastrol induced ROS generation and promoted JNK phosphorylation and activation, leading to the initiation of autophagy. The autophagy triggered by celastrol-induced ROS accumulation contributed to apoptosis in osteosarcoma cells. In vivo, celastrol could significantly suppress the growth of human osteosarcoma xenograft at doses of 1 and 2 mg/kg, with 5.7 and 9% of weight loss in mice, respectively, suggesting that a high dose of celastrol had some side effects [124]. In addition, another study found that celastrol initiated autophagy via the ROS/JNK signaling pathway, but that celastrol-mediated autophagy promoted the survival and inhibited apoptosis in glioma cells [125]. This discrepancy might be due to the cell-type-dependent effect of celastrol.

### 4.2. Curcumin

Curcumin, a chemical class of polyphenols, is the active ingredients obtained from the extract of *Curcuma longa* L. (also named turmeric belonging to *Zingiberaceae* family). Turmeric, commonly regarded as a condiment and pigment, has been used for traditional Chinese medicine in Asia for thousands of years [171,172]. Although curcumin has some unfavorable galenic properties such as variable solubility and low bioavailability, its medicinal value has attracted much attention. Recently, it has been demonstrated that curcumin displays extraordinary anti-cancer effects in colorectal, leukemia, cervical, prostate, and breast cancer. For example, curcumin suppressed the breast cancer cell proliferation and invasion by regulating NF-κB and Nrf2, inhibiting the human epidermal growth factor receptor 2 (HER2) and EGFR signaling, or modulating miRNAs [173]. To increase the solubility and bioavailability, researchers encapsulated curcumin in natural excipients gum acacia (GA) microspheres and then conjugated folic acid on the surface of these curcumin-GA microspheres. The curcumin-loaded GA microspheres could obviously reduce tumor volume and caused no significant toxicity in a triple negative breast cancer animal model [174]. Recently, curcumin has been proven to scavenge electrons and ROS in multiple in vitro models, which give the credit to its phenolic analogs serving as electron receptors to destabilize ROS [175,176]. In addition, there are many reports demonstrating that curcumin induced or inhibited autophagy by employing different molecular mechanisms in diversified in vitro or in vivo models [177]. Noteworthy, Lee et al. found that treatment of colon cancer cells with curcumin led to an increase of LC3 conversion, decrease in p62 levels, and ultimately, cell death, which could be almost completely blocked by the antioxidant N-acetylcystein (NAC) [178]. Similar results were further obtained in curcumin-treated cervical cancer [139]. These reports indicate that curcumin induces autophagy-mediated cell death by promoting ROS accumulation. However, the mechanisms underlying ROS-induced autophagy and the role of curcumin-induced autophagy in cell death remain largely elusive.

### 4.3. Allicin

In 1893, Fernie’s group firstly reported the benefits of functional foods, such as garlic and onion, for human health [179]. Garlic has been used as a spice since ancient times, and a wide range of ancient tests described its extensive use in the treatment of various diseases. Allicin is the main bioactive ingredient in garlic and is responsible for the typical taste and smell of crushed garlic. It was proved that allicin is a thioester of sulfenic acid generated by an enzymatic reaction after injury of the garlic tissue [180]. Till now, allicin has been shown to display extensive biological actions and pharmacological functions, including antimicrobial, anti-inflammatory, anti-cancer, and immune-modulatory activities [181,182]. Allicin exhibits superior anti-cancer effects for a variety of cancers, covering leukemia, lymphoma, cholangiocarcinoma, gastric, hepatic, breast, lung, prostatic, renal, colon, endometrial, cervical, and bladder cancer [183]. On a molecular level, allicin can regulate many signaling pathways such as p53, STAT3, JNK, Nrf2, and MAPK. In addition, allicin suppresses cancer cell proliferation by modulating the interplay between ROS and autophagy. Namita’s group found allicin treatment led to the accumulation of ROS, the activation of MAPK/JNK, and the induction of autophagy in non small cell lung cancer (NSCLC) cells. The use of ROS eliminators could rescue allicin-induced MAPK/JNK activation and autophagy induction, indicating that allicin can induce MAPK/JNK-mediated autophagy by promoting ROS accumulation [157]. Meanwhile, authors further interpreted that ROS-mediated autophagy at a low dose of allicin is cytoprotective, whereas a high dose of allicin leads to autophagic cell death. In addition, it was found that allicin could considerably induce oxidative stress and autophagy to suppress osteosarcoma growth via inactivating the MALAT1-miR-376a-Wnt/β-catenin axis, or promote ROS production and p53-dependent autophagy in live cancer, but it is not clear whether autophagy was regulated by ROS [156,184].

### 4.4. Erianin

Erianin is a low-molecular-weight bibenzyl natural product extracted from *Dendrobium chrysotoxum Lindl*, a traditional Chinese medicine with extensive clinical applications due to its tonic, analgesic, astringent, and anti-inflammatory properties [185]. Modern pharmacological studies have shown that erianin have various biological activities, such as anti-cancer activity. For instance, erianin could induce apoptosis by reducing Bcl-2 expression and activating caspase signaling in T47D human breast cancer cells, or via inhibiting ERK1/2ERKsignaling and activating p53 in cervical cancer cells [186,187]. Meanwhile, treatment with erianin could arrest the cell cycle at the G2/M phase by regulating the expression of p53, p27, and p21 in cancers, including gastric cancer, colon cancer, and osteosarcoma. Cancer migration and angiogenesis could also be inhibited by erianin through modulating the expression of ERα, p-ERK1/2, MMP2, MMP9, TIMP1/2, COX-2, HIF-1α, and IL-6, or the activation of JAK2/STAT3 [187]. Moreover, erianin was found to promote ROS accumulation, JNK activation, apoptosis, and autophagy. Pretreatment with antioxidant NAC evidently reversed these consequences, implying that erianin induces autophagy through the ROS-dependent JNK/c-Jun pathway [126].

### 4.5. Chrysin

Chrysin (5,7-di-OH-flavone) is a natural dietary flavonoid compound that exists abundantly many plant extracts such as honey, blue passion flower (*Passiflora caerulea*), *Radix scutellariae*, and *Pleurotus ostreatus*. Though the therapeutic application of chrysin in the clinic is still incipient on account of its low bioavailability and absorption, the diverse pharmacological activities of chrysin have attracted a lot of attention. Chrysin improved glucose and lipid metabolism disorders by activating the AMPK/PI3K/AKT signaling pathway in insulin-resistant HepG2 Cells [188]. In an animal model of agitated depression induced by olfactory bulbectomy (OB), chrysin had the ability to attenuate the depressant-like behavior and hippocampal dysfunction depressant-like behavior, similarly to fluoxetine [189]. Increasing evidence also demonstrates that chrysin exhibits anti-cancer effects [190,191]. In terms of molecular regulation, ROS was generated following treatment with chrysin to induced ER stress and apoptosis in bladder cancer cells [192]. Chrysin-treated anaplastic thyroid cancer (ATC) tumors revealed increased protein levels of Notch1 and Hes1 (hairy/enhancer of split 1), and activated Notch1 might induce cleaved Poly ADP ribose polymerase (PARP) protein, indicating that apoptosis could be induced to inhibit cancer cells [193]. A study also found that chrysin promoted the production of intracellular ROS, which inactivated AKT/mTOR signaling pathway and induced autophagy in endometrial cancer. Inhibition of ROS-mediated autophagy further sensitized cancer cells to chrysin treatment, suggesting that chrysin induces ROS-mediated cytoprotective autophagy in endometrial cancer [141].

### 4.6. Isoorientin

Isoorientin [160] is a glucoside composed of luteolin with a β-d-glucosyl residue at C-6, which was first discovered in rooibos by Koeppen’s group in 1962 [194]. Meanwhile, accumulating evidence has revealed that isoorientin is a kind of flavonoid and is sourced from a wide array of edible plants, such as fenugreek seeds and buckwheat groats. Isoorientin has a series of pharmacological activities including antioxidant, anti-inflammatory, and anti-cancer activities [195]. It has been proven that isoorientin could suppress epithelial-to-mesenchymal processes and cancer stem-cell-like features by inhibiting the Wnt/β-catenin/STAT3 pathway in oral squamous cell carcinoma [196]. Treatment with isoorientin could induce apoptosis in lung cancer cells through the ROS-mediated MAPK/STAT3/NF-κB signaling pathway [197]. Li Y et al. demonstrated that isoorientin could also inhibit the PI3K/AKT, p53, and NF-κB signal pathway and activate the JNK and p38 pathway by increasing ROS levels, leading to the induction of autophagy and apoptosis, thereby inhibiting the proliferation of liver cancer cells [160].

### 4.7. Capsaicin

Pepper, one of the most popular vegetable crops in the world, is rich in minerals, vitamins, carotenoids, and capsaicinoids. One of the important compounds extracted from pepper is capsaicin (trans-8-methyl-*N*-vanillyl-6-nonenamide), which is responsible for the irritant and burning consequences [198]. During the last couple of years, increasing evidence has demonstrated that capsaicin is a biologically active phytochemical that possesses multifarious pharmacological activities. It possesses an intensive pain relief function and early studies established fundamental concepts in pain neurobiology using capsaicin. The capsaicin 8% was proven to reduce spontaneous pain, mechanical allodynia, and cold-evoked pain in chemotherapy-induced peripheral neuropathy (CIPN) [199,200]. Moreover, it has anti-oxidant, anti-diabetes, weight loss, blood pressure falling, and anti-cancer activities [201]. The anti-cancer activity of capsaicin has been observed in various cancers. Mechanistically, treatment with capsaicin could inhibit breast cancer proliferation by down-regulating the NF-κB pathway [202], and attenuated bladder cancer cell migration via SIRT1 inhibition to enhance cortactin and β-catenin acetylation [203]. Moreover, capsaicin can also induce apoptosis and cell cycle arrest through the suppression of EGFR and activation of the AMPK pathway [204]. In addition, capsaicin can also induce autophagy and ROS to inhibit the growth of cancer cells. For example, capsaicin inhibited PI3K/AKT pathways by inducing ROS generation to block autophagy, thus contributing to the inhibition of proliferation in prostate cancer cells [146]. In another study, capsaicin was reported to induce autophagy by enhancing ULK1 acetylation through reducing the deacetylase activity of the tumor-associated NADH oxidase-sirtuin 1 (tNOX-SIRT1) complex. Meanwhile, capsaicin provoked the generation of ROS, which may play an important role in capsaicin-mediated suppression of SIRT1 activation and subsequent autophagy induction in melanoma cancer cells [205].

### 4.8. Pristimerin

Pristimerin is a quinone methide triterpenoid that was firstly extracted from *Pristimerae indica* in 1951 [206]. Emerging evidence has demonstrated that pristimerin is a biologically active phytochemical possessing various biological effects, including anti-inflammatory, anti-bacterial, insecticidal, anti-viral, anti-fungal, and anti-cancer effects. Pristimerin could inhibit Wnt/β-catenin signaling by activation of GSK3β, thereby suppressing Wnt target gene expression to suppress the growth of colon cancer cells [207]. In addition, ROS accumulation could be caused by pristimerin treatment to induce ER stress, resulting in the activation of JNK signaling and induction of apoptosis in colon cancer cells [208]. Increasing evidence shows that pristimerin exerts an anti-cancer effect through modulating the relationship between autophagy and ROS. In detail, pristimerin could activate JNK signaling by promoting ROS accumulation in chronic myeloid leukemia (CML) cells. ROS-mediated JNK activation then triggered autophagy and apoptosis to inhibit cancer growth [127]. A similar effect of pristimerin on ROS and autophagy was also recapitulated in breast cancer cells [128].

### 4.9. Neohesperidin

Neohesperidin is a flavanone glycoside isolated from citrus fruits and is commonly used in the food industry, such as in the synthesis of neohesperidin dihydrochalcone (NHDC). Neohesperidin administration could attenuate obesity, low-grade inflammation, and insulin resistance by reversing high-fat diet (HFD)-induced intestinal microbiota dysbiosis in mice fed a HFD [209]. The combination of neohesperidin dihydrochalcone and empagliflozin inhibited oxidative stress liberation, inflammatory mediator production, and apoptotic reactions to protect against methotrexate (MTX)-induced renal injury [210]. Other pharmacological activities of neohesperidin have also been observed, including cardiovascular protection and suppression of osteoclast differentiation activity [211]. Moreover, neohesperidin has been proven to block the growth of cancer cells generally by inducing cell cycle arrest and promoting apoptosis. Notably, neohesperidin-treated cells exhibited higher levels of ROS, leading to the activation of JNK signaling and subsequent induction of autophagy, thereby provoking apoptosis in osteosarcoma cells [130].

### 4.10. Polyphyllins

The rhizome of *Paris polyphylla*, named Chong-lou in traditional Chinese medicine, has been used to make various trademarked herbal regimes, such as “Yunnan Baiyao” and “Jidesheng Sheyaopian”. Chong-lou is applied to treat parotitis, mastitis, snakebite, fractures, throat, abscess convulsion, and various human malignancies [212]. It mainly contains steroidal saponins, phytoecdysones, phytosterols, and flavonoids, among which steroidal saponins are the major bioactive components [213]. Increasing evidence has shown that polyphyllins, a class of steroidal saponins, regulated redox homeostasis and autophagy to inhibit cancer cell growth. For example, poplyphylla VI (PPVI) treatment led to an elevation of ROS levels, which triggered autophagic cell death by inactivating the mTOR signaling pathway in NSCLC [214]. It has also been reported that PPVI could promote ROS generation to activate the JNK signaling pathway, resulting in the induction of autophagy and apoptosis in glioma and osteosarcoma cells [131,215]. In addition to PPVI, polyphyllin VII (PPVII) was found to promote ROS accumulation to inhibit AKT/mTOR signaling, thereby inducing cytotoxic autophagy in glioma cells [216].

### 4.11. Magnoflorine

Magnoflorine is a vital quaternary aporphine alkaloid extracted from various genera of flowering plant families, such as *Menispermaceae*, *Ranunculaceae,* and *Magnoliaceae* [217]. Increasing studies have revealed that magnoflorine exhibits various pharmacological activities, including anti-inflammatory, antioxidant, anti-diabetic, antifungal, hypotensive, and immunomodulatory activities. In recent years, the anti-cancer effect of magnoflrine has aroused much attention. Magnoflorine has been shown to activate p38 and inhibit AKT/mTOR signaling pathways to induce autophagy, which further exacerbated doxorubicin-induced apoptosis, thereby increasing the sensitivity of breast cancer cells to doxorubicin [218]. In gastric cancer, magnoflorine could promote autophagic cell death through the generation of ROS and suppression of AKT/mTOR signaling [134].

### 4.12. Baicalin

The roots of *S.baicalensis Georgi*, *Scutellaria rivularia wall*, *Scutellaria galericulata,* and *Scutellaria lateflora* L. belong to Chinese herbal medicine and were once used to cure dysentery, hyperlipidemia, hypertension, atherosclerosis, and respiratory ailments in ancient China. Baicalin, and its aglycone, baicalein, is a flavonoid and bioactive phytochemical extracted from these roots. Baicalin and baicalein have been proven to exhibit numerous pharmacological activities. Baicalin could protect LPS-induced blood-brain barrier damage and decrease ROS generation by activating the Nrf2 pathway [219]. Baicalin treatment could effectively attenuate acetaminophen (APAP)-induced liver injury by enhancing the ERK signaling pathway, so also has hepatoprotective properties. Moreover, baicalin is a cardioprotective, antiviral, neuroprotective, and antithrombotic agent [220,221]. As early as 1994, researchers found that baicalin had the ability to inhibit liver cancer growth [222]. Nowadays, it has been demonstrated that the growth of various cancers, such as lung, breast, bladder, and colon cancers, could also be suppressed by baicalin [223]. For example, baicalin was able to induce ROS accumulation to inhibit the PI3K/AKT/mTOR signaling, thereby triggering cytotoxic autophagy in human osteosarcoma cells [136]. In another study, baicalin was loaded with folic acid-modified albumin nanoparticles (FA-BSANPs/BA) to improve its biocompatibility and prolong its circulation time in vivo. The FA-BSANPs/BA nanoparticles were found to elevate ROS levels, inactivate AKT/mTOR signaling, and induce cytotoxic autophagy and apoptosis in breast cancer [137]. These results demonstrate that baicalin can regulate ROS-mediated autophagy in cancer.

### 4.13. Bigelovin

Bigelovin is a sesquiterpene lactone extracted from *Inula helianthus-aquatica* and has been identified as a selective retinoid X receptor α agonist. Several studies have demonstrated that bigelovin exhibited anti-cancer activities [224,225]. Bigelovin was evidenced to inhibit the growth of many cancers, including lung cancer, glioma, gastric cancer, and leukemia. For example, bigelovin treatment promoted ROS generation, leading to the induction of apoptosis and autophagy in liver cancer cells. Inhibition of autophagy sensitized liver cancer cells to bigelovin treatment, suggesting that bigelovin-induced autophagy is cytoprotective [138]. However, whether ROS plays a role in bigelovin-induced protective autophagy requires further investigation.

### 4.14. Diosgenin

Sapogenins are a series of glycoside chemicals extracted from numerous natural products that are beneficial to health. Diosgenin is a steroidal sapogenin, which is plentiful in *Smilax china*, *Rhizoma polgonati*, *Dioscorea villosa*, *Dioscorea rhizome,* and *Trigonella foenum-graecum*, and can also be found in the *Dioscoreaceae*, *Liliaceae*, *Scrophulariaceae*, *Rhamnaceae*, *Solanaceae*, *Leguminosae*, *Amaryllidaceae*, and *Agavaceae* families. Diosgenin has been widely studied for its excellent therapeutic effect on various chronic diseases. Importantly, diosgenin has been used as a principal precursor compound for the synthesis of several steroidal drugs by pharmaceutical companies [226]. Multiple investigations suggest that diosgenin has diverse biological activities, such as antioxidant, hypolipidemic, anti-inflammatory, anti-proliferative, and hypoglycemic activities. Recently, its anti-cancer effect has drawn much attention. In a transgenic prostate cancer mouse model, diosgenin could abrogate NF-κB/STAT3 activation, and this action attenuated cancer cell growth and metastasis [227]. In addition, diosgenin could sensitize HCC and gastric cancer cells to doxorubicin and inhibitors of the enhancer of zeste homology 2 (EZH2), respectively [228,229]. Specifically, it has been shown that diosgenin can inhibit mTOR pathway by up-regulating ROS generation to induce autophagy in chronic myeloid leukemia cells, and inhibiting autophagy can enhance diosgenin-induced apoptosis [139].

### 4.15. Trichosanthin

The root tuber of *Trichosanthes kirilowii* (Gua Lou) from the family of *Cucurbitaceae* was used in traditional Chinese medicine as an abortifacient called Tian Hua Fen. Both Simiao Sun and Shizhen Li recorded that Tian Hua Fen can deal with abortion, abnormal menstruation, and retained placenta in the traditional medical book Qianjin Yifang and Compendium of Materia Medica, respectively [230,231]. The precipitated Tian Hua Fen powder was crystallized in barbitone buffer and acquired as the single protein powder of trichosanthin in 1982. Trichosanthin is a type 1 ribosome-inactivating protein (RIP), which can be activated by removing the N-terminal 23 aa signal peptide and C-terminal 19 aa peptide. Activated trichosanthin can cleave the N-glycosidic bond at adenine-4324 of 28S rRNA to intercept the protein synthesis function of the ribosomes, leading to cell death. Recently, trichosanthin was found to exhibit anti-virus activity against human immunodeficiency virus (HIV), herpes simplex virus (HSV), and hepatitis B virus (HBV) [232,233]. Besides, it has been demonstrated that trichosanthin could also inhibit the growth of cancer cells by inducing apoptosis, autophagy, and cell cycle arrest [233]. In human gastric cancer cells, Trichosanthin could induce ROS generation. Trichosanthin-mediated ROS accumulation led to increased phosphorylation of NF-κB and p53, which then inhibited cell proliferation by inducing autophagy [153]. However, how NF-κB and p53 cooperate to induce autophagy needs to be further investigated. Notably, the half-life of trichosanthin in the body is very short because it can be easily filtered and cleared by the kidney, which severely limits its application. In this regard, some studies linked trichosanthin with the albumin binding domain mutant ABD035 (abbreviated as ABD), and found that trichosanthin-ABD could bind with endogenous human serum albumin by ABD to prolong its half-life and enhance the anti-cancer effect in vivo [234].

### 4.16. Piperlongumine

*Piper longum* L. is the most famous species of the pepper family and can be used to make spices such as black pepper. Its seeds, roots, and fruits have been used as herbal medicine because of the various medicinal properties [235,236]. There are many biologically active components extracted from *Piper longum* L. such as piperlongumine. Piperlongumine is an alkaloid/amide and can also be found in other piper plants such as *Piper arborescens Roxb* and *Piper chaba Hunter.* Chatterjee and co-workers firstly identified the chemical structure of piperlongumine in the 1960s. Recently, a series of studies have shown that piperlongumine exhibits various pharmacological activities. For example, piperlongumine could mitigate hyperglycemia via rescuing pancreatic β cells, and inhibits inflammation and apoptosis by modulating the GLUT-2/4 and AKT signaling pathway in streptozotocin-induced diabetic rats [237]. Piperlongumine could also inhibit the NF-κB and MAPK signaling pathways to protect the brain against ischemic cerebral injury [236,238]. In addition, piperlongumine was found to exhibit promise anti-cancer activities in various cancers. In castration-resistant prostate cancer cells, DNA could be damaged persistently by piperlongumine treatment to inhibit proliferation, migration, and invasion of prostate cancer cells [239]. Apoptosis and cell cycle arrest can also be induced by piperlongumine in gastric, cervical, and colorectal cancer cells [240]. San-Yuan Chen et al. proved that treatment with piperlongumine activated ERK pathway to induce autophagy in biliary cancer, which can be recovered by NAC. It demonstrated that piperlongumine could induce autophagy via ROS-mediated REK signaling, but whether autophagy participates in the anti-cancer of piperlongumine requires further investigation [155].

### 4.17. Betulinic Acid

Betulinic acid is a naturally pentacyclic triterpenoid that is widely distributed in *Betula* species plants and can also be found in various species of the *Syzygium*, *Diospyros*, *genera Ziziphus,* and *Paeonia*. It is proven that BA has strong anti-HIV effects. Several derivatives possessing anti-HIV properties have been synthesized and have entered phase I/II clinical trials. Pisha et al. firstly found that betulinic acid could selectively inhibit the growth of human melanoma in 1995. From then on, its anti-tumor activity has been extensively reported. Betulinic acid can serve as a potential inducer of apoptosis or autophagy in various cancers. In human cervical cancer cells, treatment with betulinic acid could downregulate PI3K/Akt signaling by promoting ROS generation, resulting in the stimulation of mitochondrial pathway of apoptosis [241]. Besides, Yan Zhang et al. found that BA could activate the AMPK/mTOR/ULK1 pathway by increasing ROS generation to induce autophagy in human bladder cancer cells, and inhibition of ROS-mediated autophagy could restore BA-induced apoptosis [152].

### 4.18. Rg3-Enriched Red Ginseng Extract (Rg3-RGE)

*Panax ginseng*, a Chinese herbal medicine, has been applied to treat different diseases and enhance immunity for more than 2000 years. Ginseng contains various bioactive compounds, including ginsenosides, peptides, polysaccharides, mineral oils, and fatty acids [242]. Rg3-enriched red ginseng extract (Rg3-RGE) is a single ginnsenoside extracted from *panax ginseng* and is well known for its pharmacological activities, including inhibiting platelet activation and thrombus formation, anti-inflammatory, and anti-diabetic activities [243]. Increasing studies demonstrated Rg3-RGE could also inhibit the growth of cancers such as lung cancer. Rg3-RGE treatment led to mitochondrial damage and increased the levels of mitochondrial ROS in lung cancer cells. It then generated ROS-induced PINK1-Parkin mediated mitophagy to remove damaged mitochondria, suppression of which by mitophagy inhibitors promoted mitochondrial dysfunction and resultant cell death [244].

## 5. Discussion

In this review, we presented a straightforward introduction to the autophagy process and its role in cancer, explained the reciprocal regulatory relationship between ROS and autophagy, and illustrated some phytochemicals that can regulate the relationship between autophagy and ROS through different signaling pathways in cancer. What we can conclude is that ROS may activate dormant oncogenes by acting as a signaling molecule, but beyond that point, ROS also cause DNA damage to induce cancer cell death. Meanwhile, autophagy inhibits cancer formation in normal tissue while promoting tumor progression in advanced cancer. In addition, autophagy can regulate ROS levels by removing cellular oxidized components and ROS can also promote and modulate autophagy through complex mechanisms. Therefore, ROS and autophagy have diversified impact on cancer because they can exhibit changeable functions and dynamic processes as well as mutual regulation depending on cell type and microenvironment. Therefore, understanding and manipulating the relationship between ROS and autophagy is expected to increase drug sensitivity and provide new insights into cancer treatment.

Cancer treatment has been regarded as one of the most critical and important topics in clinical issues. Increased therapeutics have been developed depending on the types and stages of the cancer, and chemotherapy remains a main therapeutical strategy at present. However, drug resistance and toxic side effects markedly limit the clinical application of many existing chemotherapeutic drugs. Considering the extraordinary chemical diversity and generally acceptable toxicity, natural products are considered as a strong source for anti-cancer drug discovery. Phytochemicals are biologically active substances found in plants. Extensive studies have illustrated their anti-cancer activities in various types of cancers through multiple mechanisms, such as the modulation of autophagy and ROS. Interestingly, whether autophagy induced by phytochemical-mediated ROS leads to cancer cell survival or death may depend on the cell type. For example, autophagy induced by diosgenin-mediated ROS accumulation plays a protective role in chronic myeloid leukemia cells; the use of autophagy inhibitors can increase diosgenin-induced apoptosis. However, baicalin-induced autophagy through ROS/AKT/mTOR signaling has a harmful effect on breast cancer. Furthermore, celastrol was reported to promote apoptosis induction in osteosarcoma by ROS/JNK-mediated autophagy, whereas the celastrol-mediated ROS accumulation and autophagy induction promote the survival of glioma cells. Therefore, using antioxidants or autophagy inhibitors to suppress ROS-mediated protective autophagy may be a potential therapeutic approach for cancer treatment. However, in some cases, the use of antioxidants or autophagy inhibitors may also reduce ROS- or autophagy-induced cell death. Hence, it is necessary to have a broad understanding of the cellular events (such as tissue, cell, stage, autophagy levels, and ROS levels) occurring in different cancers, which can enable us to strictly monitor ROS, autophagy, and cancer cell death in vivo, and select more effective drug combinations for various therapies.

Many phytochemicals are in the relatively early stages of drug development, and there are several challenges and problems that need to be solved. Firstly, the assessment of most anti-cancer studies are dependent on in vitro evaluation, and drug-associated toxicity is an important barrier for currently available chemotherapeutic agents. Thus, animal studies and preclinical studies need to be conducted to verify their anti-cancer effects and side effects, as well as the optimal concentration or dose for use. During the process of animal studies, the control group must be conducted strictly to evaluate the outcome of experimental conditions. Secondly, the low solubility and poor bioavailability of natural extracts limit their further clinical research and development. The development of biopolymeric materials for gene and agent transport has become a promising area in biomedical research. Encapsulating the drug in nanoparticles can modulate the speed of drug release, increase the permeability of biofilm, change the distribution in the body, and improve the drug bioavailability. Therefore, the studies of phytochemicals delivery and nanoparticles are expected to solve these problems.

## Figures and Tables

**Table 1:** Mechanisms of action of phytochemicals that can regulate autophagy and ROS.

Phytochemicals	Mechanism of Autophagy Regulation by ROS	Cancer types	References
Carnosol	ROS/Beclin1	Triple negative breast cancer	[123]
Celastrol	ROS/JNK; ROS/PI3K/Akt/mTOR	Osteosarcoma; Glioma	[124] [125]
Erianin	ROS/JNK	Osteosarcoma	[126]
Pristimerin	ROS/JNK	Chronic myeloid leukemia; Breast cancer	[127,128]
Juglanin	ROS/JNK	Breast cancer	[129]
Neohesperidin	ROS/JNK	Osteosarcoma	[130]
Polyphyllin VI	ROS/JNK	Osteosarcoma	[131]
Polyphyllin VII	ROS/JNK	Osteosarcoma	[132]
Actein	ROS/JNK	Bladder cancer	[133]
Magnoflorine	ROS/JNK	Gastric cancer	[134]
Ampelopsin	ROS/JNK	Glioma	[135]
Baicalin	ROS/AMPK/mTOR/ULK1	Breast cancer; Osteosarcoma	[136,137]
Bigelovin	ROS/PI3K/Akt/mTOR	Liver cancer	[138]
Diosgenin	ROS/PI3K/Akt/mTOR	Chronic myeloid leukemia cells	[139]
Beta-Lapachone	ROS/PI3K/Akt/mTOR	Nasopharyngeal cancer cells	[140]
Chrysin	ROS/PI3K/Akt/mTOR	Endometrial cancer	[141]
6-Methoxydihydrosanguinarine	ROS/PI3K/Akt/mTOR	Breast cancer	[142]
Neferine	ROS/PI3K/Akt/mTOR	Lung cancer	[143]
Eldecalcitol	ROS/PI3K/Akt/mTOR	Osteosarcoma	[144]
Momordin Ic	ROS/PI3K/Akt/mTOR	Hepatoblastoma cancer	[145]
Capsaicin	ROS/PI3K/Akt/mTOR	Prostate cancer	[146]
Artesunate	ROS/AMPK/mTOR/ULK1	Bladder cancer;	[147]
Dendrobium	ROS/AMPK/mTOR/ULK1	Colon cancer	[148]
Daphnetin	ROS/AMPK/mTOR/ULK1	Ovarian cancer	[149]
beta-Elemene	ROS/AMPK/mTOR/ULK1	Colorectal cancer	[150]
Britannin	ROS/AMPK/mTOR/ULK1	Liver cancer	[151]
Betulinic acid	ROS/AMPK/mTOR/ULK1	Bladder cancer	[152]
Trichosanthin	ROS/NF-κB	Gastric cancer	[153]
Cryptotanshinone	ROS/NF-κB	Colon cancer	[154]
Piperlongumine	ROS/ERK	Biliary cancer	[155]
Allicin	ROS/p53	Osteosarcoma; Lung Cancer; Thyroid cancer; Liver Cancer	[156,157,158,159]
Isoorientin	ROS/p53; ROS/PI3K/AKT; ROS/JNK	Liver cancer	[160]
Alantolactone	ROS/AKT/PINK1/mitophagy	Hepatoblastoma cancer	[103]
Sanguinarine	ROS/mitophagy	Hepatocellular carcinoma	[161]
TEOA	ROS/mitophagy	Colorectal cancer	[162]
Corilagin	ROS/autophagy	Gastric cancer	[163]
Curcumin	ROS/autophagy	Cervical cancer	[164]
Anthocyanin	ROS/autophagy	Liver cancer	[165]

## Data Availability

Not applicable.
